# A Qualitative Work System Analysis Using a Human Factors Engineering Approach to Evaluate Environmental Cleaning in Veterans’ Affairs Hospitals

**DOI:** 10.1017/ash.2021.121

**Published:** 2021-07-29

**Authors:** Linda McKinley, Cassie Goedken, Erin Balkenende, Stacey Hockett Sherlock, Heather Reisinger, Mary Jo Knobloch, Eli Perencevich, Nasia Safdar

## Abstract

**Background:** Environmental cleaning is important in the interruption of pathogen transmission and subsequent infection. Although recent initiatives have targeted cleaning of high-touch surfaces and incorporated audit-and-feedback monitoring of cleaning practices, practice variations exist and compliance is still reportedly low. Evaluation of human factors influencing variations in cleaning practices can be valuable in developing interventions, leading to standardized practices and improved compliance. We conducted a work system analysis using a human-factors engineering framework [the Systems Engineering Initiative for Patient Safety (SEIPS) model] to identify barriers and facilitators to current environmental cleaning practices within Veterans’ Affairs hospitals. **Methods:** We conducted semistructured interviews with key stakeholders (ie, environmental staff, nursing, and infection preventionists) at 3 VA facilities across acute-care and long-term care settings. Interviews were conducted among 18 healthcare workers, audio recorded, and transcribed verbatim. Transcripts were analyzed for thematic content within the SEIPS constructs (ie, person, environment, organization, tasks, and tools). **Results:** Within the SEIPS domain ‘person,’ we found that many environment service (EVS) staff were veterans and were highly motivated to serve fellow veterans, especially to prevent them from acquiring infections. However, the hiring of service members as EVS staff comes with significant hurdles that affect staffing. Within the domain of ‘environment’, EVS staff reported rooms that were either occupied by the patient or were multibed, were more difficult to clean. Conversely, they reported that it was easier to clean in settings where the patient was more likely to be out of bed (eg, long-term care residents). Patient flow and/or movement greatly influenced workload within the ‘organizational’ domain. Workload also changed by patient population and setting (eg, the longer the stay or more critical the patient), increased their workload. EVS staff felt that staffing consistency and experience improved cleaning practices. Within the ‘task’ domain, EVS staff were motivated for cleaning high-touch surfaces; however, knowledge of these surfaces varied. Finally, within the ‘tool’ domain, most EVS staff described having effective cleaning products; however, sometimes in limited supply. Most sites reported some form of monitoring of their cleaning process; however, there was variation in type and frequency. **Conclusions:** Human-factors analysis identified barriers to and facilitators of cleaning compliance. Incorporating environmental cleaning practices that address barriers and facilitators identified may facilitate standardized cleaning of environmental surfaces. Standardized procedures for cleaning multibed rooms and environmental surfaces surrounding occupied beds may improve cleaning compliance. Future research should evaluate standardized cleaning procedures or bundles that incorporate these best practices and steps to overcoming barriers and pilot feasibility.

**Funding:** No

**Disclosures:** None

